# Parabolic–Gaussian Double Quantum Wells under a Nonresonant Intense Laser Field

**DOI:** 10.3390/nano13081360

**Published:** 2023-04-14

**Authors:** Esin Kasapoglu, Melike Behiye Yücel, Carlos A. Duque

**Affiliations:** 1 Department of Physics, Faculty of Science, Sivas Cumhuriyet University, 58140 Sivas, Türkiye; ekasap@cumhuriyet.edu.tr; 2 Department of Physics, Faculty of Science, Akdeniz University, 07058 Antalya, Türkiye; myucel@akdeniz.edu.tr; 3 Grupo de Materia Condensada-UdeA, Instituto de Física, Facultad de Ciencias Exactas y Naturales, Universidad de Antioquia UdeA, Calle 70 No. 52-21, Medellín 050003, Colombia

**Keywords:** parabolic–Gaussian potential, double quantum well, intense laser field

## Abstract

In this paper, we investigate the electronic and optical properties of an electron in both symmetric and asymmetric double quantum wells that consist of a harmonic potential with an internal Gaussian barrier under a nonresonant intense laser field. The electronic structure was obtained by using the two-dimensional diagonalization method. To calculate the linear and nonlinear absorption, and refractive index coefficients, a combination of the standard density matrix formalism and the perturbation expansion method was used. The obtained results show that the electronic and thereby optical properties of the considered parabolic–Gaussian double quantum wells could be adjusted to obtain a suitable response to specific aims with parameter alterations such as well and barrier width, well depth, barrier height, and interwell coupling, in addition to the applied nonresonant intense laser field.

## 1. Introduction

It is very important to construct the ideal potential energy function of diatomic and/or polyatomic molecules. The greater the number of parameters in the potential energy function, the better the fit with the experimental results. The potential energy function proposed by Morse in 1929 [1] was used to study diatomic and polyatomic molecules [2]. In addition, potential energy functions such as Rosen–Mors, Manning–Rosen, Schiöberg, Tietz, and modified Lennard–Jones that are used for diatomic molecules were also established and successfully used to directly fit the experimental results to some diatomic molecules [3,4,5,6]. Double quantum wells such as quartic [7], Konwent [8], Razavy [9], and Manning [10] were also some proposed potential energy functions to probe diatomic molecules.

Quantum wells are widely applied to light-emitting devices and lasers. Quantum-well-based light-emitting diodes (QW-LEDs) use a quantum-well structure to improve their performance. There are several types of QW-LEDs, including single-quantum-well (SQW), multiple-quantum-well (MQW), and superlattice LEDs. QW-LEDs have several advantages over traditional LEDs. Because the quantum well is so thin, it restricts the motion of electrons and holes, which improves the efficiency of the recombination process that produces light, resulting in brighter and more efficient LEDs. QW-LEDs also have a narrower emission spectrum than that of traditional LEDs, rendering them useful in applications where specific light colors are required, such as in traffic lights and electronic displays. They are also used in high-speed optical communication systems where their narrow spectral width and high modulation speed are important. The tuneability of the inherent band gap of III nitride materials renders them attractive for white light-emitting diodes (WLEDs) that are considered the next-generation solid-state lighting sources. In this context, GaN-based LEDs have attracted considerable interest due to their usefulness in disinfection, automotive front lighting, solid-state lighting, and full-color displays, low energy consumption, long operational lifetime, and a broad spectral range spanning from the ultraviolet to the red wavelengths [11,12]. GaAs-based LEDs typically operate in the infrared region of the electromagnetic spectrum. GaAs materials generally have a wavelength of about 940 nm. They are semiconductors that are used in various optoelectronic applications, such as TV remotes, cameras, medical applications, and remote-sensing, inducing, and intelligent systems [13].

Double quantum wells (DQWs) separated by a barrier layer are the best semiconductor heterostructural samples that demonstrate quantum tunneling. For a sufficiently wide barrier, the coupling between the wells is eliminated, and the DQW turns into two separate and independent single wells. That is, the wells become decoupled, and the energies had twofold degeneracy. If the barrier is thin enough, the coupling between the quantum wells (QWs) starts via tunneling in the barrier. Tunneling through the barrier splits the twofold degenerate single states into symmetric and antisymmetric doublet states. The disappearance of degeneracy in the energy levels depends on the respective potential parameters and the applied external fields. Since the nonlinear optical properties of semiconductor QWs mostly depend on the asymmetry of the confinement potential, when examining the optical properties of heterostructures such as quantum wells, wires, and dots, either their asymmetrical shapes are selected or an electric field is applied to symmetrical shapes [14,15,16,17,18,19,20,21,22]. Advanced high-power tuneable laser sources have motivated studies on the interaction of a high-frequency intense laser field (ILF) with carriers in semiconductors [23]. In the presence of an ILF, different quantum structures were intensively researched [24,25,26,27,28,29,30,31,32,33]. The electronic and optical properties of engineered band-edge QWs could be significantly changed by adjusting the amplitude of the ILF due to variations in the size and shape of the potential induced by radiation. Furthermore, experimental studies in the presence of an ILF are available [34,35]. The potential in this study had been used to calculate eigenstates for the inversion of hydrogen in ammonia [36], and for the proton transfer between two water molecules [37]. Ammonia inversion is an important problem in chemistry that has been studied by many researchers. The potential for the vibration causing inversion is mostly considered parabolic with an internal potential barrier to inhibit [38].

The potential for umbrella inversion in ammonia refers to the energy required to rotate the three hydrogen atoms around the nitrogen atom, causing the molecule to invert its shape, like an umbrella. The potential energy diagram for the umbrella inversion shows the relationship between the potential energy and the hydrogen atoms’ rotation angle. The potential energy diagram for umbrella inversion in ammonia had a double-well shape, which means that there are two energetically favorable positions for hydrogen atoms: one above and another below the nitrogen atom. These two positions correspond to the two lobes of the potential energy curve, separated by a barrier at the top of the curve. The required energy to overcome this barrier and switch the hydrogen atoms from one lobe of the potential energy curve to the other is relatively small, with a typical barrier height of about 24 kJ/mol (0.249 eV per particle) [36]. This means that umbrella inversion could occur millions of times per second at room temperature, rendering it an important phenomenon in studying ammonia and similar molecules. Understanding the potential energy of umbrella inversion in ammonia is essential for studying its spectroscopic and chemical properties, and modeling its behavior in various chemical and biological processes.

In this study, we examined the optical properties of an electron in both symmetrical and asymmetrical DQWs formed with the sum of Gaussian barrier and parabolic well potentials under an ILF. The Gaussian barrier between the parabolic wells created a grouping by adjoining the nearest onto symmetric and antisymmetric states. All energy states increased due to the presence of the barrier, but the energies of the antisymmetric states increased less than symmetric ones since they had a node that was not in symmetrical states.

This work is arranged as follows: the theory is presented in Section 2, the results and discussion are outlined in Section 3, and the conclusions are given in Section 4.

## 2. Theory

The Hamiltonian of an electron within effective mass approximation is as follows:

(1) $$H=\frac{{\vec{p}}^{2}}{2\;m^{*}}+V\left(z\right)\;,$$

where $\vec{p}$ and $m^{*}$ are the momentum and effective mass of the electron, respectively; V(z) is the parabolic–Gaussian double quantum well (P–G DQW) potential. In the case without ILF, it is as follows [39]:

(2) $$V\left(z\right)=V_{o}\;\left[A_{1}{\left(z/k\right)}^{2}+A_{2}e^{-{\left(\frac{z}{k}-z_{o}\right)}^{2}}\right]\;,$$

where $V_{o}$ is the QW depth, and *k* and $z_{o}$ are width and asymmetric parameters, respectively. $A_{1}$ and $A_{2}$ are the potential constants that adjust the coupling between wells, barrier height, and well depth and width. The potentials defined in [36,37] were adjusted for this study.

In the presence of the ILF, the potential in Equation (2) was defined as the laser-dressed potential [29,40,41,42,43,44] as below:

(3) $$V\left(z,\alpha_{o}\right)=\frac{\omega_{o}}{2\pi }\int_{0}^{2\pi /\omega_{o}}V\left(z+\alpha_{o}\sin\left(\omega_{o}\;t\right)\right)dt\;,$$

where $\alpha_{o}=eA_{o}/m^{*}\omega_{o}$, $A_{o}$ and $\omega_{o}$ are the laser-dressing parameter, the magnitude of the vector potential, and the angular frequency of the nonresonant ILF, respectively. To obtain the eigenvalues and eigenfunctions of the electron confined within the P–G DQW potential under ILF, we used a diagonalization method with the selection of a wave function that expanded on a base of trigonometric orthonormal functions [45].

For transitions between any two allowed energy levels, absorption coefficients (ACs) and refractive index changes (RICs), including linear and nonlinear terms, are found as follows, respectively [18,29,42,43,44,46,47,48].

(4) $$\beta^{\left(1\right)}\left(\omega \right)=\sqrt{\frac{\mu_{o}}{\varepsilon_{r}}}\frac{|M_{ij}{|}^{2}\sigma_{\nu }\hbar \omega \Gamma_{ij}}{{\left(E_{ij}-\hbar \omega \right)}^{2}+{\left(\hbar \Gamma_{ij}\right)}^{2}}\;,$$

(5) $$\begin{array}{cc} \beta^{\left(3\right)}\left(\omega ,I\right)= & -2\sqrt{\frac{\mu_{o}}{\varepsilon_{r}}}\left(\frac{I}{\varepsilon_{o}n_{r}c}\right)\frac{|M_{ij}{|}^{4}\sigma_{\nu }\hbar \omega \Gamma_{ij}}{{\left[{\left(E_{ij}-\hbar \omega \right)}^{2}+{\left(\hbar \Gamma_{ij}\right)}^{2}\right]}^{2}}\;\\ \; & \times \left[1-\frac{|M_{jj}-M_{ii}{|}^{2}}{|2M_{ij}{|}^{2}}\frac{{\left(E_{ij}-\hbar \omega \right)}^{2}-{\left(\hbar \Gamma_{ij}\right)}^{2}+2E_{ij}\left(E_{ij}-\hbar \omega \right)}{E_{ij}^{2}+{\left(\hbar \Gamma_{ij}\right)}^{2}}\right]\;, \end{array}$$

(6) $$\beta \left(\omega \right)=\beta^{\left(1\right)}\left(\omega \right)+\beta^{\left(3\right)}\left(\omega ,I\right)\;,$$

(7) $$\frac{\Delta n^{\left(1\right)}\left(\omega \right)}{n_{r}}=\frac{\sigma_{v}\;{\left|M_{ij}\right|}^{2}}{2\;\varepsilon_{o}\;n_{r}^{2}}\;\;\frac{E_{ij}-\hbar \;\omega }{{\left(E_{ij}-\hbar \;\omega \right)}^{2}+{\left(\hbar \;\Gamma_{io}\right)}^{2}}\;,$$

(8) $$\begin{array}{c} \frac{\Delta n^{\left(3\right)}\left(\omega ,I\right)}{n_{r}}=-\frac{\mu_{o}\;c\;I\;\sigma_{v}\;{\left|M_{ij}\right|}^{2}}{4\;\varepsilon_{o}\;n_{r}^{3}}\;\;\frac{E_{ij}-\hbar \;\omega }{{\left[{\left(E_{ij}-\hbar \;\omega \right)}^{2}+{\left(\hbar \;\Gamma_{ij}\right)}^{2}\right]}^{2}}\;\\ \times \left[4\;|M_{ij}{|}^{2}-\frac{|M_{jj}-M_{ii}{|}^{2}}{E_{ij}^{2}+{\left(\hbar \;\Gamma_{ij}\right)}^{2}}\;\;\left\{E_{ij}\;\left(E_{ij}-\hbar \;\omega \right)-{\left(\hbar \;\Gamma_{ij}\right)}^{2}-{\left(\hbar \;\Gamma_{ij}\right)}^{2}\frac{\left(2\;E_{ij}-\hbar \;\omega \right)}{\left(E_{ij}-\hbar \;\omega \right)}\right\}\right]\;, \end{array}$$

(9) $$\frac{\Delta n(\omega ,I)}{n_{r}}=\frac{\Delta n^{\left(1\right)}\left(\omega \right)}{n_{r}}+\frac{\Delta n^{\left(3\right)}\left(\omega ,I\right)}{n_{r}}\;,$$

where $\varepsilon_{r}=n_{r}^{2}\;\varepsilon_{o}$ is the real permittivity, $\varepsilon_{o}$ and $\mu_{o}$ are the permittivity and permeability of the vacuum, respectively, $\sigma_{\nu }$ is the carrier density, $E_{ij}=E_{j}-E_{i}$ is the energy difference, $M_{ij}=\langle \psi_{i}\left|e\;z\right|\psi_{j}\rangle$, and (i,j=1,2) is the dipole matrix element (DME) for eigenstates $\psi_{i}$ and $\psi_{j}$ for *z*-polarized radiation. $\Gamma_{ij}$ is the relaxation rate, *c* is the speed of light, and *I* is the photon intensity with the ω angular frequency.

## 3. Results and Discussion

The values of the physical parameters were $\varepsilon_{o}=12.58$, $m^{*}=0.067m_{o}$ (where $m_{o}$ is the free electron mass), $V_{o}=228$ meV, $n_{r}=3.2$, $\Gamma_{12}={\left(0.2\times 10^{-12}\right)}^{-1}$ s${}^{-1}$, $\mu_{o}=4\pi \times 10^{-7}$ Hm${}^{-1}$, $\sigma_{\nu }=3.0\times 10^{22}$ m${}^{-3}$, and $I=5.0\times 10^{8}$ W/m${}^{2}$.

In this study, $z_{o}$ was the asymmetric parameter; for $z_{o}=0$ ($z_{o}\neq 0)$, the structure was symmetric (asymmetric). In this context, P–G SDQW and P–G ADQW mean symmetric and asymmetric cases, respectively.

**Figure 1:**
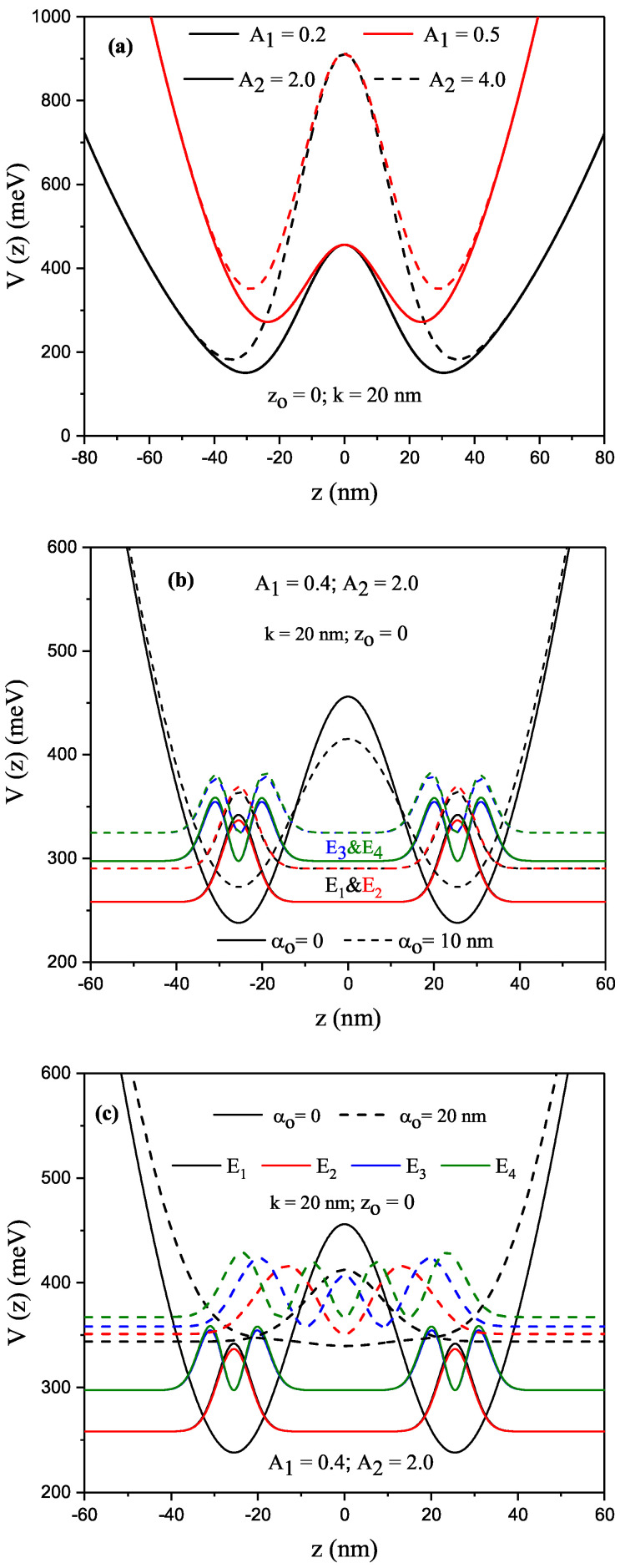
Parabolic–Gaussian symmetric DQW ($z_{o}=0$) potential for a constant value of *k*(=20 nm) versus the *z*-coordinate. Parabolic–Gaussian symmetric DQW, solid (dashed) curves are for $A_{2}=2.0$ $\left(A_{2}=4.0\right)$ and black (red) curves $A_{1}=0.2$ $\left(A_{1}=0.5\right)$ (**a**), Parabolic–Gaussian symmetric DQW confinement profiles and squared modulus of the wave-functions corresponding to the first 4 energy levels for $\alpha_{o}=0$ (solid curves) and $\alpha_{o}=10\;\mathrm{nm}$ (dashed curves) (**b**), and Parabolic–Gaussian symmetric DQW potential and squared modulus of the wave-functions corresponding to the first 4 energy levels for $\alpha_{o}=0$ (solid curves) and $\alpha_{o}=20\;\mathrm{nm}$ (dashed curves) (**c**). Results are for k=20 nm, $A_{1}=0.4$ and $A_{2}=2.0$.

Variations in the shape of P–G SDQW potential versus the z coordinate in the absence of the nonresonant ILF for different structural parameters are given in Figure 1a–c. Figure 1a shows that parameter $A_{1}$ caused a shift towards higher energies in the confinement potential, and a decrease in both effective width (well width + barrier width) and barrier height. Parameter $A_{2}$ created a decrement in the effective well width, and an increment in the height and width of the barrier. Figure 1b,c present variations in the potential of different structural parameters and the squared modulus of the wave functions of the four energy levels, with each located at the energy level of the electron confined within the P–G SDQW under nonresonant ILF versus the *z* coordinate for $\alpha_{o}=0$ (solid curves) and $\alpha_{o}=10\;\mathrm{nm}$ (dashed curves) and of $\alpha_{o}=0$ (solid curves) and $\alpha_{o}=20\;\mathrm{nm}$ (dashed curves). Figure 1b shows that the energies had double-fold degeneracy in the cases without and with ILF. With the effect of ILF, the energies of the electron increased, the confinement potential shifted towards higher energies, and the barrier height decreased. Figure 1c shows that P–G SDQW became a single large QW since the ILF caused the potential barrier to disappear for $\alpha_{o}=20\;\mathrm{nm}$.

**Figure 2:**
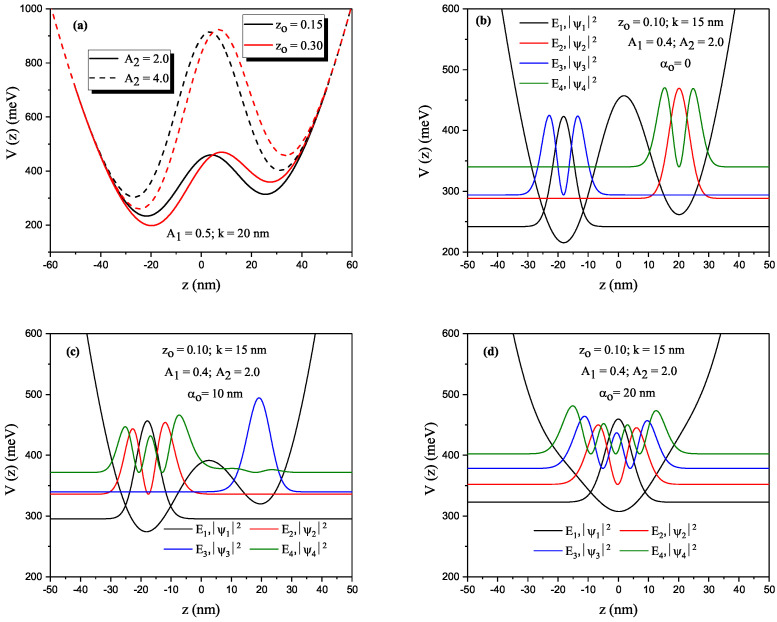
(**a**) Parabolic–Gaussian asymmetric DQW confinement profiles for the parameters of k=20 nm, $A_{1}=0.5$, $A_{2}=2.0$ (solid curves) and $A_{2}=4.0$ (dashed curves) and for two different $z_{o}$ values, black (red) curve is for $z_{o}=0.15$ $\left(z_{o}=0.30\right)$, parabolic–Gaussian asymmetric DQW potential and squared modulus of the wave functions corresponding to the first four energy levels for (**b**) $\alpha_{o}=0$, (**c**) $\alpha_{o}=10\;\mathrm{nm}$, and (**d**) $\alpha_{o}=20\;\mathrm{nm}$. The results in Figure 2b–d are for $z_{o}=0.10$, k=15 nm, $A_{1}=0.4$, $A_{2}=2.0$.

Figure 2a–d show effects of different asymmetric and structural parameters, and the ILF on the potential and energies of the electron confined within the P–G ADQW. Figure 2a shows the changes in the shape of the confinement potential for a single $A_{1}$ value with two different $z_{o}$ and $A_{2}$ parameters. $z_{o}$ caused the widths of two wells to be different from each other, and the QW in the right-hand side became narrower and shifted upward relative to the left-hand side. This situation, which occurs with the asymmetric parameter in the absence of an electric field, can be made asymmetrical by applying an electric field to the SDQW. Figure 2b–d show the variations in the potential and the squared modulus of the wave functions of the first four energy levels, each other located at its own energy level of the electron confined within the P–G ADQW with width k=15 nm versus the *z* coordinate for $\alpha_{o}=0$, $\alpha_{o}=10\;\mathrm{nm}$ and $\alpha_{o}=20\;\mathrm{nm}$. In the absence of the ILF, electrons that had $E_{1}$ and $E_{3}$ energies were located in the left well, and the others (electrons with $E_{2}$ and $E_{4}$ energies) were in the right well. For $\alpha_{o}=10\;\mathrm{nm}$, while the electron with $E_{3}$ energy remained entirely in the right well, the electron with $E_{4}$ energy electron penetrated the left well, but there was also a small probability of finding the right well. For $\alpha_{o}=20\;\mathrm{nm}$, P–G ADQW became a single parabolic QW because the ILF caused the potential barrier to disappear.

**Figure 3:**
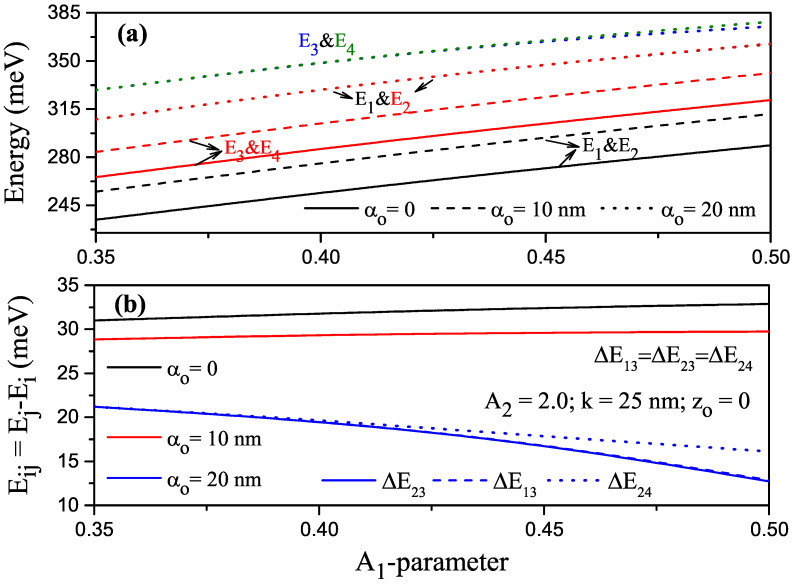
For k=25 nm, $A_{2}=2.0$, $z_{o}=0$, (**a**) changes in the energies of the electron confined within parabolic–Gaussian symmetric DQW under the intense laser field versus parameter $A_{1}$; (**b**) variation of energy differences between related levels versus the $A_{1}$ parameter. Solid, dashed, and dotted curves are for $\alpha_{o}=0$, $\alpha_{o}=10\;\mathrm{nm}$, and $\alpha_{o}=20\;\mathrm{nm}$, respectively.

For k=25 nm, $A_{2}=2.0$ and $z_{o}=0$, the variation in the first four lowest energy levels and energy differences between these levels of the electron confined within P–G SDQW under the ILF as a function of the $A_{1}$ parameter are given in Figure 3a,b, where solid, dashed, and dotted curves are for $\alpha_{o}=0$, $\alpha_{o}=10\;\mathrm{nm}$ and $\alpha_{o}=20\;\mathrm{nm}$, respectively. Energies were the increasing functions of parameters $A_{1}$ and ILF; energy differences between some levels, such as $E_{1}$ and $E_{3}$, $E_{2}$ and $E_{3}$, and $E_{2}$ and $E_{4}$ were equal to each other ($E_{13}=E_{23}=E_{24}$) for $\alpha_{o}=0$ and $\alpha_{o}=10\;\mathrm{nm}$ since the energies were twofold degenerate. Energy differences increased the functions of parameter $A_{1}$ for these ILF values.For $\alpha_{o}=20\;\mathrm{nm}$, since the structure became a single QW after a certain $A_{1}$ value with the effect of ILF, the degeneracy of the energies disappeared, and the energy differences decreased with increasing $A_{1}$ values for the above-mentioned levels.

**Figure 4:**
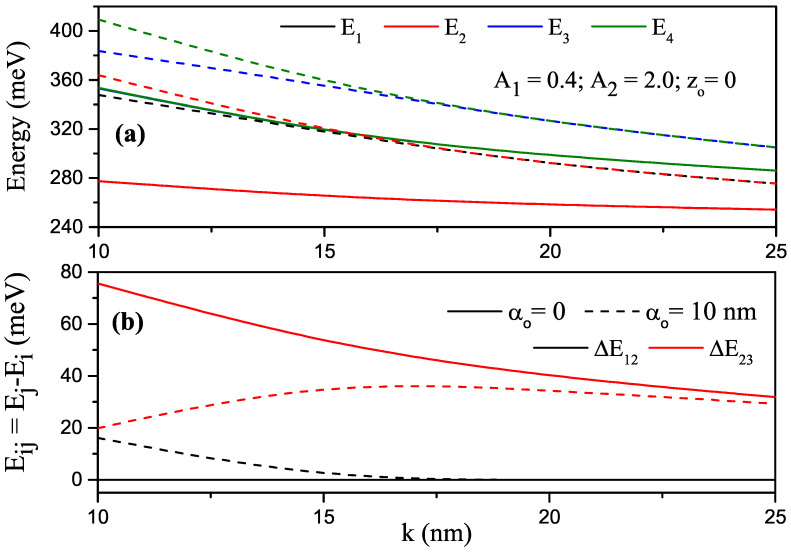
For $A_{1}=0.4$, $A_{2}=2.0$ and $z_{o}=0$, (**a**) changes in the energies of the electron confined within parabolic–Gaussian symmetric DQW under the intense laser field versus the *k* parameter; (**b**) variation of energy differences between related levels versus the *k* parameter. Solid and dashed curves are for $\alpha_{o}=0$ and $\alpha_{o}=10\;\mathrm{nm}$, respectively.

In the cases without and with ILF, for k=25 nm, $A_{1}=0.4$, $A_{2}=2.0$ and $z_{o}=0$, the variations in the first four lowest energy levels and energy differences between two levels of the electron confined within P–G SDQW as a function of the *k* width parameter were obtained as shown in Figure 4a,b, where solid and dashed curves are for $\alpha_{o}=0$ and $\alpha_{o}=10\;\mathrm{nm}$, respectively. Since the increase in *k* parameter weakened the confinement, the electronic energies were decreased. Twofold degenerate energies in the absence of ILF split with the effect of ILF, and after a certain *k* value, the energies became doubly degenerate again because, as *k* increased, electrons that passed into the weak confinement regime had lower energies than that of the potential barrier. Figure 4b, shows that $E_{12}=0$ since the ground and first excited states were twofold degenerate, and $E_{23}$ decreased with increasing *k*-value in the absence of ILF. In the presence of the ILF, $E_{12}$ decreased, $E_{23}$ increased up to a certain *k* value, $E_{12}$ became zero, and $E_{23}$ started to decrease again.

**Figure 5:**
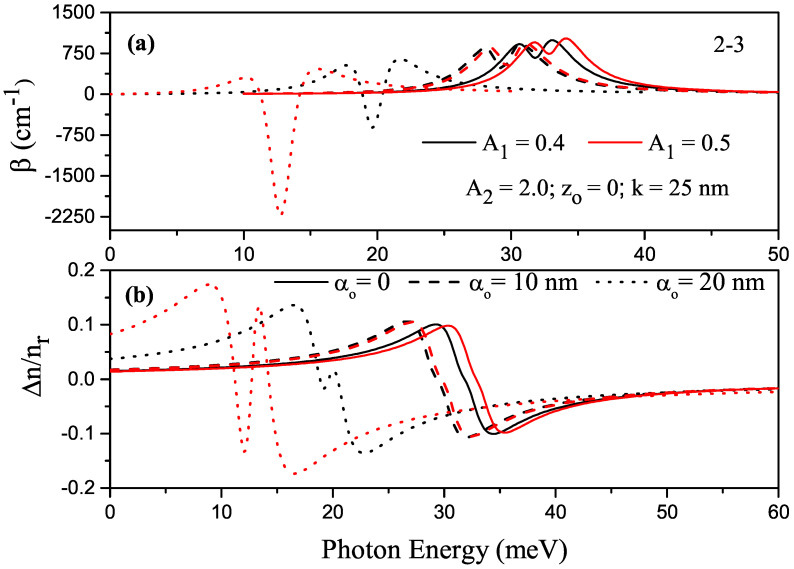
For k=25 nm, $A_{2}=2.0$ and $z_{o}=0$, (**a**) variation in the total absorption coefficient versus the photon energy corresponding to the (2-3) transition in parabolic–Gaussian symmetric DQW; (**b**) variation in the total refractive index versus the photon energy, where black (red) curves are for $A_{1}=0.4$ $\left(A_{1}=0.5\right)$ and solid, dashed, and dotted curves are for $\alpha_{o}=0$, $\alpha_{o}=10\;\mathrm{nm}$, and $\alpha_{o}=20\;\mathrm{nm}$, respectively.

For k=25 nm, $A_{2}=2.0$ and $z_{o}=0$, the variations in total ACs and total RICs as a function of the incident photon energy corresponding to the (2-3) transition in P–G SDQW are given in Figure 5a,b, where black (red) curves are for $A_{1}=0.4$ $\left(A_{1}=0.5\right)$, and solid, dashed, and dotted curves are for $\alpha_{o}=0$, $\alpha_{o}=10\;\mathrm{nm}$, and $\alpha_{o}=20\;\mathrm{nm}$, respectively. In Figure 3a,b, where there was no ILF, the considered levels had twofold degeneracy $(E_{1}=E_{2}$ and $E_{3}=E_{4})$, and only transition (2-3) was allowed. When the intensity of ILF increased, a red shift was observed in the peak positions of both AC and RIC, depending on the change in energy difference; a blue shift was also observed with an increase in parameter $A_{1}$.

**Figure 6:**
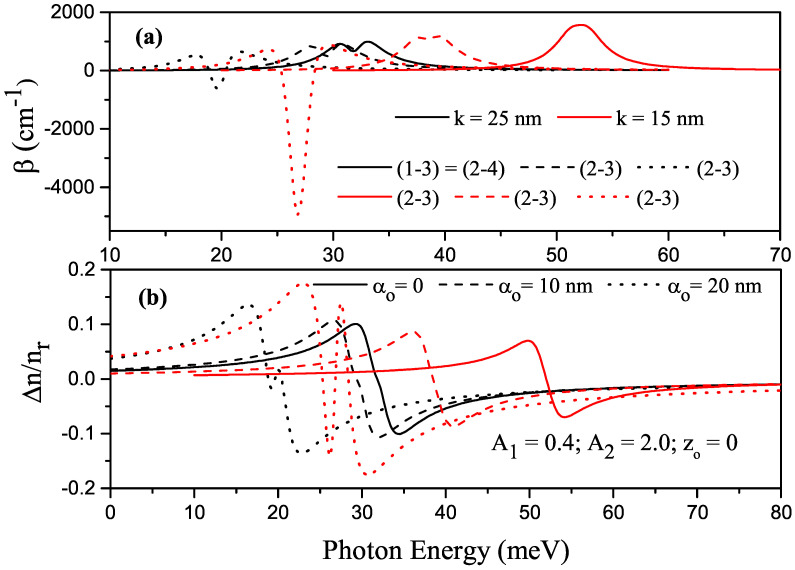
For the transitions between energy levels in parabolic–Gaussian symmetric DQW with parameters $z_{o}=0$, $A_{1}=0.4$ and $A_{2}=2.0$, (**a**) change in total absorption coefficients versus the photon energy; (**b**) variation in the total refractive index versus the photon energy, where black (red) curves are for k=25 nm (k=15 nm) and solid, dashed, and dotted curves are for $\alpha_{o}=0$, $\alpha_{o}=10\;\mathrm{nm}$, and $\alpha_{o}=20\;\mathrm{nm}$, respectively.

Figure 6a,b show the variations in total ACs and RICs as a function of the incident photon energy for the allowed transitions between energy levels in P–G SDQW with parameters $\alpha_{o}=0$, $A_{1}=0.4$ and $A_{2}=2.0$, where black (red) curves are for k=25 nm (k=15 nm), and solid, dashed, and dotted curves are for $\alpha_{o}=0$, $\alpha_{o}=10\;\mathrm{nm}$, and $\alpha_{o}=20\;\mathrm{nm}$, respectively.

In the large *k* values (black curves), AC shifted to red since the energies decreased due to the increasing well width. The total AC peaks belonging to the transitions between the first and third (or second and fourth) levels had the same energy difference but different wave functions divided into two peaks called the bleaching effect. In this study, the bleaching effect was observed only for the large *k* value because the nonlinear term was greater than that of the linear term in the small *k* value. In the absence (presence) of ILF, (1-3) transition is allowed (forbidden). For small *k* values (red curves), ACs belonging to the allowed transitions shifted towards the blue, and the (2-3) transition was allowed. For both *k* values, the changes in the position and amplitude of the total RIC were in good agreement with the total AC.

In the symmetrical structures ($z_{o}=0$), the diagonal matrix elements with respect to the parities of the wave functions were zero $\left(M_{jj}=M_{ii}=0\right)$. That is, the DMEs for the transitions between the odd and even states (i.e., 1-3 or 2-4) were zero (these transitions were forbidden) because the envelope functions of these levels had the same parity. However, if the structural symmetry is disturbed, such transitions become free.

**Figure 7:**
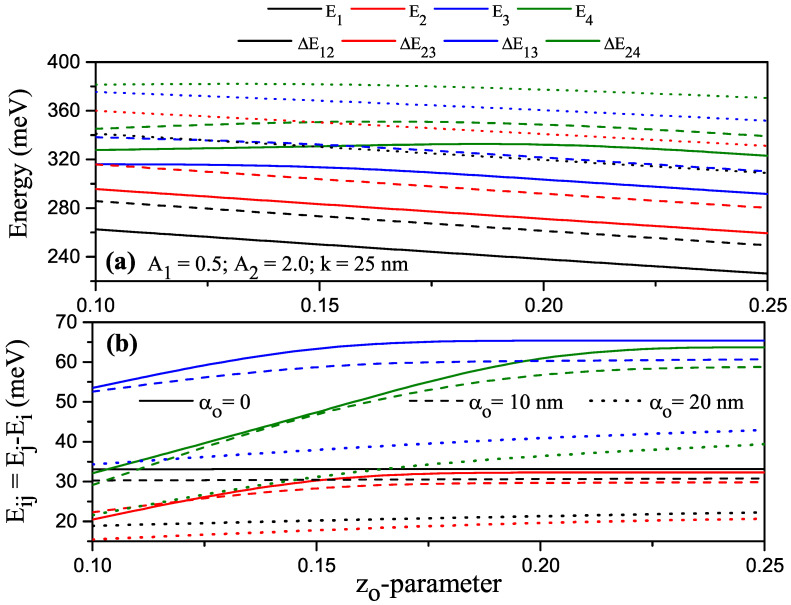
For $A_{1}=0.5$ and $A_{2}=2.0$ and k=25 nm, (**a**) variations in the first four lowest energy levels of the electron confined within parabolic–Gaussian asymmetric DQW under an intense laser field with respect to the $z_{o}$ parameter; (**b**) variation in energy differences between some levels versus the $z_{o}$ parameter. Solid, dashed, and dotted curves are for $\alpha_{o}=0$, $\alpha_{o}=10\;\mathrm{nm}$, and $\alpha_{o}=20\;\mathrm{nm}$, respectively.

For the electron in P–G ADQW that had parameters $A_{1}=0.5$, $A_{2}=2.0$ and k=25 nm under the ILF, variations in the energy levels and energy differences between some levels versus the $z_{o}$ parameter are given in Figure 7a,b; solid, dashed, and dotted curves are for $\alpha_{o}=0$, $\alpha_{o}=10\;\mathrm{nm}$, and $\alpha_{o}=20\;\mathrm{nm}$, respectively. Except for the fourth energy level, the three lower energy levels were decreasing functions of the asymmetric parameter for $\alpha_{o}=0$ and $\alpha_{o}=10\;\mathrm{nm}$. For $z_{o}=0.10$, the electrons in the first, second, and fourth levels were localized in the left well (LW), and the electrons in the third level were localized in the right well (RW). When $z_{o}=0.15$, the energy of the electron in the fourth level increased because it was localized inside the RW, while the electron energies that were localized in the LW decreased. At larger $z_{o}$ values, the electron was in the LW for all levels, since P–G ADQW bends just as if an electric field is applied in the growth direction via the $z_{o}$ effect. Thanks to asymmetric parameter $z_{o}$, P–G ADQW completely turned into a single well. For $\alpha_{o}=20\;\mathrm{nm}$, as the $z_{o}$ value increased, the lower-energy electrons were found in the LW, and those in the upper level were in a large single well (no barrier) since ILF caused the barrier to disappear. Therefore, while the energy difference between the two lowest levels was almost constant for all $z_{o}$ values, the energy difference increased with $z_{o}$ for electrons at the higher levels, and when the barrier disappeared completely from the value of $z_{o}>0.20$, the energy difference of these levels also started to become constant. ILF decreased both energies and energy differences due to the reasons mentioned above.

**Figure 8:**
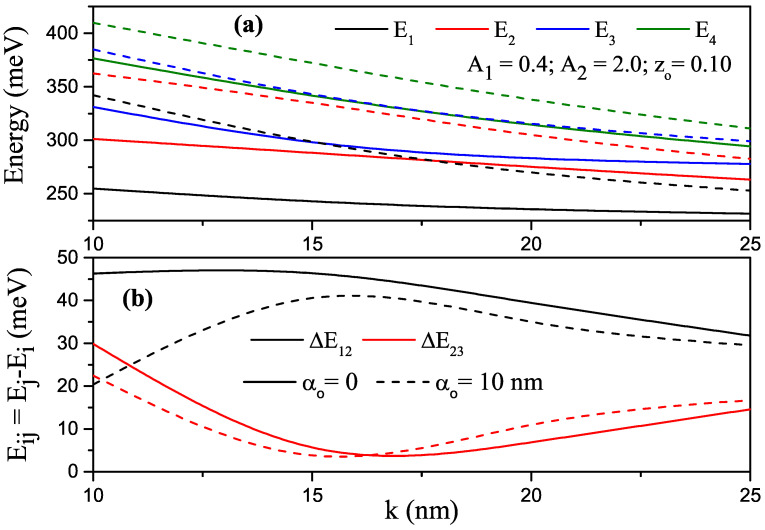
For $A_{1}=0.4$ and $A_{2}=2.0$ and $z_{o}=0.10$, (**a**) changes of the first four lowest energy levels of electron confined within parabolic–Gaussian asymmetric DQW under the intense laser field versus the *k*-parameter; (**b**) variation in energy differences between some levels versus the *k* parameter. Solid and dashed curves are for $\alpha_{o}=0$ and $\alpha_{o}=10\;\mathrm{nm}$, respectively.

Figure 8a,b shows the same regulation as that in Figure 4a,b, but the results are for the P–G ADQW corresponding to the value of asymmetric parameter $z_{o}=0.10$. The reasons are explained above. All energies were decreasing functions of the *k* parameter in the absence and presence of the ILF. In the presence of the ILF, energy values were greater than those of the case without ILF. As ILF increased, ADQW turned into a large single QW. The energy difference between these levels decreased (increased) if electrons in the related levels were localized in different (the same) wells. For example, in the absence of ILF and at small *k* values, electrons with $E_{1}$ and $E_{3}$ energies were localized in the LW, and the electron with $E_{2}$ energy was localized in the RW. As *k* increased, electrons with $E_{1}$ and $E_{2}$ energies were localized in the left well, and an electron with $E_{3}$ energy was localized in the RW. With the effect of ILF, the energy difference increased, since the electrons at all levels localized in the large single well.

**Figure 9:**
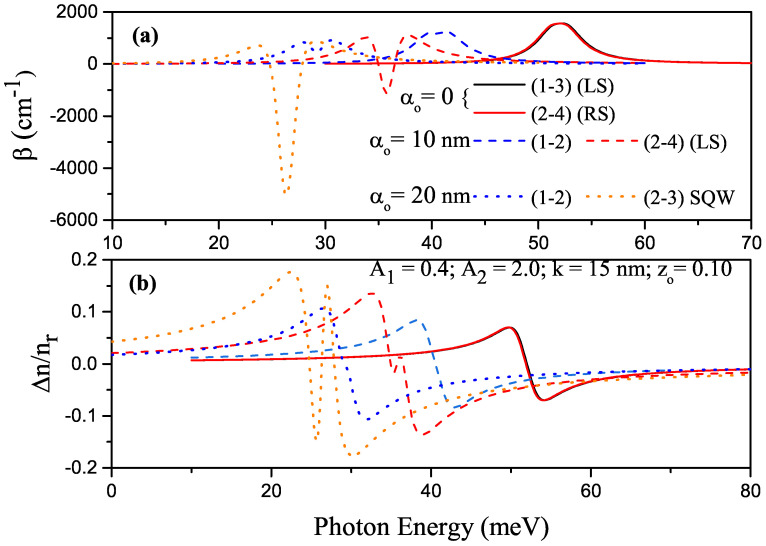
For some transitions in parabolic–Gaussian asymmetric DQW with k=15 nm, $z_{o}=0.10$, $A_{1}=0.4$ and $A_{2}=2.0$. (**a**) Variation in total absorption coefficients versus photon energy; (**b**) variation in total refractive index versus photon energy. Solid, dashed, and dotted curves are for $\alpha_{o}=0$, $\alpha_{o}=10\;\mathrm{nm}$, and $\alpha_{o}=20\;\mathrm{nm}$, respectively.

**Figure 10:**
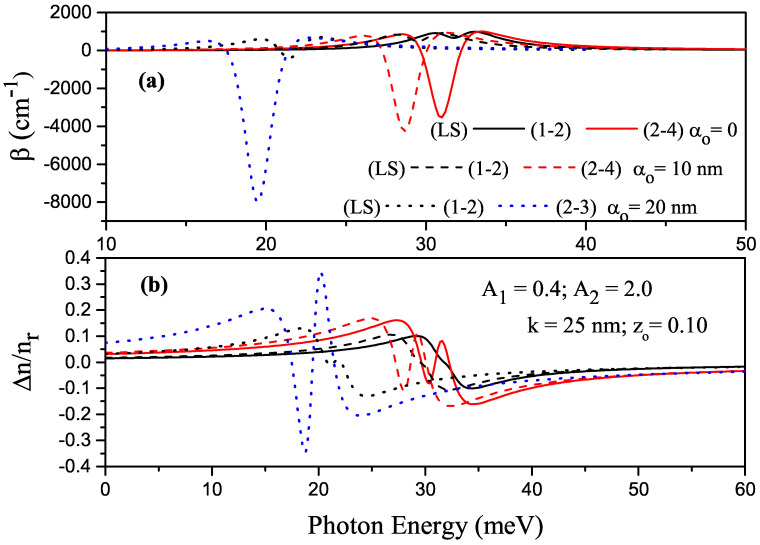
For some transitions in parabolic–Gaussian asymmetric DQW with k=25 nm, $z_{o}=0.10$, $A_{1}=0.4$ and $A_{2}=2.0$. (**a**) Variation in the total absorption coefficients concerning photon energy; (**b**) variation in total refractive index concerning photon energy. Solid, dashed, and dotted curves are for $\alpha_{o}=0$, $\alpha_{o}=10\;\mathrm{nm}$, and $\alpha_{o}=20\;\mathrm{nm}$, respectively.

For some transitions between the energy levels in P–G ADQW with the parameters of k=15 nm, $z_{o}=0.10$, $A_{1}=0.4$ and $A_{2}=2.0$, the variations in total ACs and RICs versus the photon energy are given in Figure 9a,b, respectively,. where solid, dashed, and dotted curves are for $\alpha_{o}=0$, $\alpha_{o}=10\;\mathrm{nm}$, and $\alpha_{o}=20\;\mathrm{nm}$, respectively. RS and LS indicate that the electron was on the right- or left-hand side of the well at both energy levels where the transition occurred. If there was no explanation, P–G ADQW turned into a single QW with the effect of ILF. Since the structure was asymmetrical, all transitions between all considered levels were allowed. When electrons of any two different energy levels were localized in different wells, the transition was not observed because the overlap integral $\left(I_{O}=\langle \psi_{i}|\psi_{j}\rangle \right)$ was zero or very small for the transitions between these levels. Figure 10a,b had the same regulation as that in Figure 9a,b, but the results are for k=25 nm. Changes in the positions and magnitudes of the AC and RIC are generally explained with $E_{ij}$ and $M_{ij}$, respectively. In this context, as in Figure 5a,b and Figure 6a,b, the following explanations also apply to Figure 9a,b and Figure 10a,b.

Resonance conditions for ACs in Equations (4) and (5) are given with $\hbar \;\omega_{\max}=\sqrt{{\left(E_{ij}-\hbar \omega \right)}^{2}+{\left(\hbar \Gamma_{ij}\right)}^{2}}$ and $\beta_{\max}^{\left(1\right)}\left(\omega \right)$. The maximal value of the linear AC in Equation (4) was directly proportional to the factor of $|M_{ij}{|}^{2}\left(E_{ij}+\sqrt{{\left(E_{ij}-\hbar \omega \right)}^{2}+{\left(\hbar \Gamma_{ij}\right)}^{2}}\right)$.$E_{ij}$ is the effective term on the positions of the ACs and absorption peak shift towards the smaller (greater) photon energies as the transition energy decreased (increased). The minimal value for $\beta^{\left(3\right)}\left(\omega ,I\right)$ is $\hbar \omega_{\min}=\frac{1}{3}\left(E_{ij}+\sqrt{{\left(4E_{ij}\right)}^{2}+3{\left(\hbar \Gamma_{ij}\right)}^{2}}\right)$. The $\beta^{\left(3\right)}\left(\omega ,I\right)$ term depends on parameters such as *I* light intensity (*I* was constant in this study), $|M_{ij}{|}^{4}$, and $E_{ij}$.

Further, the locations of the maximal and minimal values for the linear RIC, ${\left(\Delta n^{\left(1\right)}/n_{r}\right)}_{\max}$ and ${\left(\Delta n^{\left(1\right)}/n_{r}\right)}_{\min}$ for resonance conditions $\hbar \omega_{\max(\min)}=E_{ij}\pm \hbar \Gamma_{ij}$ were proportional to $+|M_{ij}{|}^{2}$ and $-|M_{ij}{|}^{2}$, respectively. Similarly, the locations of the maximal and minimal values of third-order nonlinear RIC-${\left(\Delta n^{\left(3\right)}/n_{r}\right)}_{\max}$ and ${\left(\Delta n^{\left(3\right)}/n_{r}\right)}_{\min}$ for the resonance conditions $\hbar \omega_{\max\;\left(\min\right)}=E_{ij}\mp \frac{1}{\sqrt{3}}\hbar \Gamma_{ij}$, were proportional to $+|M_{ij}{|}^{4}$ and $-|M_{ij}{|}^{4}$, respectively. Extreme points of the linear and nonlinear RICs were symmetrically positioned with respect to $\hbar \omega =E_{ij}$ [33]. In this context, the analyses of the locations and magnitudes of the total ACs and RICs were consistent with the results of Figure 5a,b, Figure 6a,b, Figure 9a,b, and Figure 10a,b.

## 4. Conclusions

Parabolic–Gaussian double quantum wells are a useful model to describe diatomic molecules. In this study, we examined the electronic and optical properties of an electron confined in both symmetrical and asymmetrical parabolic–Gaussian double quantum wells under an ILF by using GaAs/GaAlAs band parameters.

The split in energies due to factors such as well and barrier width, barrier height, asymmetric parameters, and intense laser fields caused transitions between the greater number of levels due to the increasing inter-sub-band energy states. The more observed peaks in the absorption spectrum give a wide degree of freedom in optoelectronic device design.

ILF reduced the interior barrier height, so the energies gradually approached the values of a wide single quantum well with increasing ILF. Asymmetric parameter $z_{o}$ created an electric-field effect when applied to the structure in the growth direction. In this context, the $z_{o}$ parameter is useful for simulating electric-field effects; thus, it manipulates the selection rules of DMEs and satisfies the emergence of the new transitions that are forbidden in symmetrical structures.

## Data Availability

No new data were created or analyzed in this study. Data sharing is not applicable to this article.
